# A Presentation of Twiddler's Syndrome with Underlying Ratchet Mechanism

**DOI:** 10.7759/cureus.4060

**Published:** 2019-02-13

**Authors:** Hassaan Iftikhar, Maryam Saleem, Muhammad Nadeem, John Caplan, Anand Kaji

**Affiliations:** 1 Internal Medicine, Seton Hall University-St. Francis Medical Center, Trenton, USA; 2 Internal Medicine, Waterbury Hospital, Waterbury, USA; 3 Cardiology, Seton Hall University-St. Francis Medical Center, Trenton, USA

**Keywords:** twiddler syndrome, reel syndrome, ratchet syndrome, pacemaker, lead dislodgement, aicd

## Abstract

Twiddler's syndrome, reel syndrome, and ratchet phenomenon are rare causes of pacemaker lead displacement. The presentation of Twiddler's syndrome with underlying ratchet mechanism is quite rarely reported in literature. In this case report we present a 63-year-old male with a history of non-ischemic cardiomyopathy who had his biventricular implantable cardioverter defibrillator leads dislodged and presented as sub-acute exacerbation of heart failure. This case highlights the underlying mechanism of twiddler's syndrome, its clinical presentation, management, and prevention.

## Introduction

Twiddler' syndrome, reel syndrome, and ratchet syndrome are rare yet dangerous phenomena of pacemaker lead dislodgement. Twiddler's syndrome was first reported by Bayliss [1]. In this case report, we describe an interesting case of pacemaker lead dislodgement by unconscious manipulation of the device pocket by a patient who presented eight months after device installation in a setting of sub-acute heart failure exacerbation.

## Case presentation

**Figure 1:**
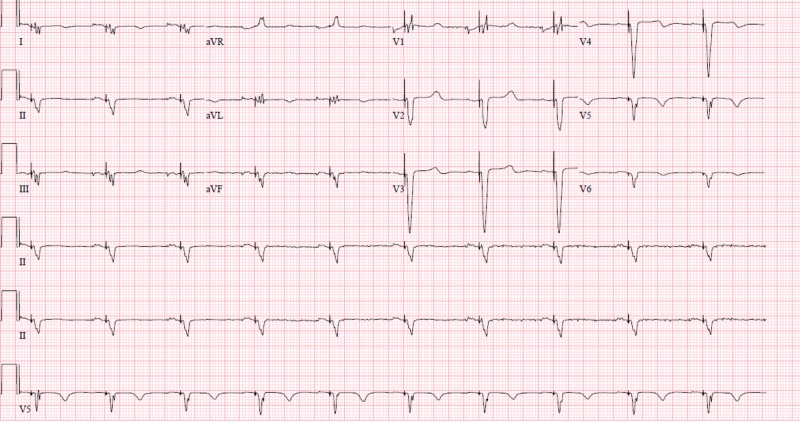
Biventricular pacing observed.

**Figure 2:**
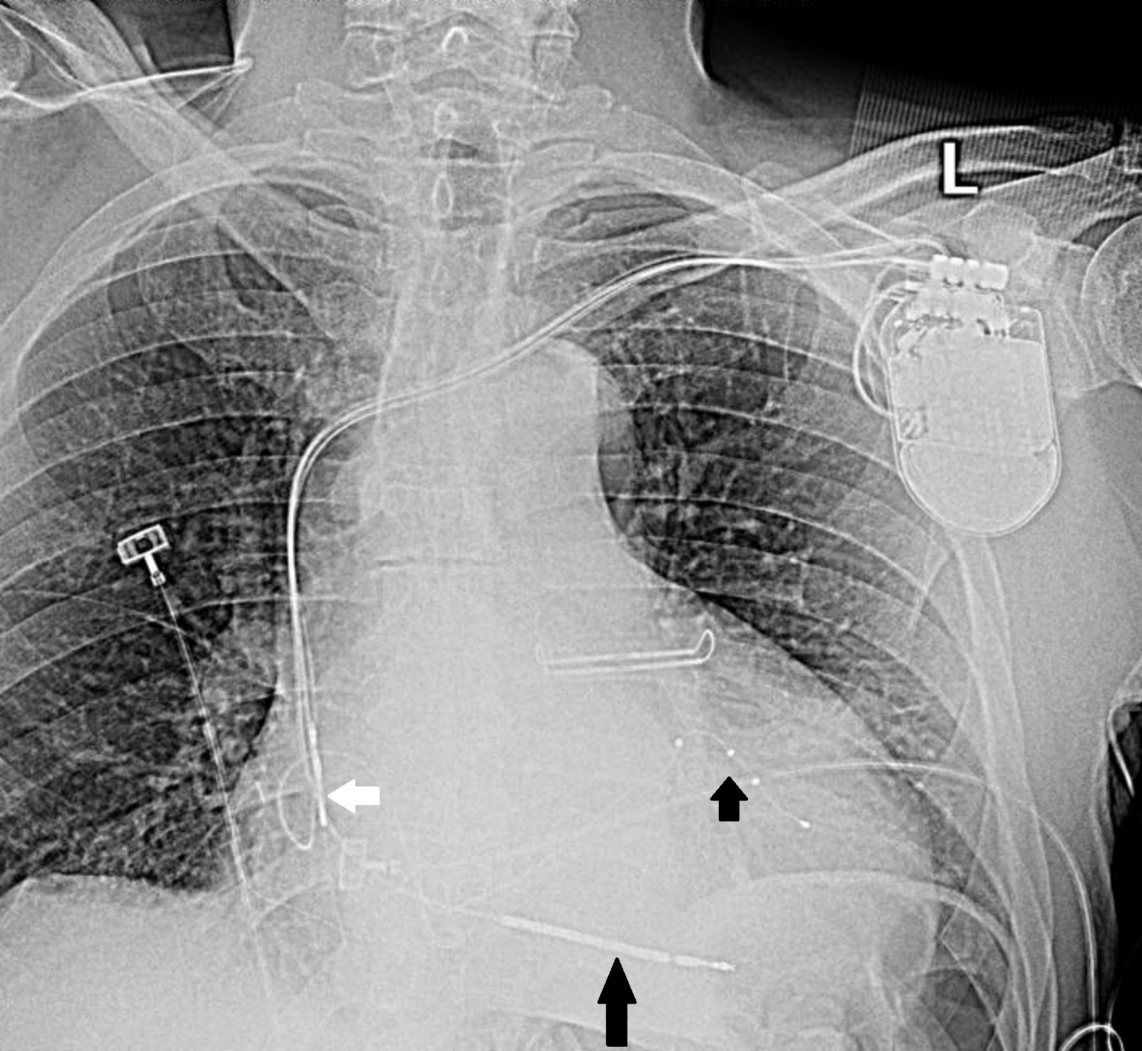
White arrow shows atrial lead, long black arrow points to defibrillator lead, and short black arrow shows left coronary sinus lead.

A 63-year-old male with a past medical history of non-ischemic cardiomyopathy (ejection fraction 10%-15%), hypertension, chronic kidney disease stage G3A, and severe mitral valve regurgitation status post valve repair initially presented for an upgradation of his biventricular pacemaker to biventricular implantable cardioverter defibrillator (BIV-ICD) and replacement of non-functional right atrial lead. The pacemaker pocket was exposed and identified and the atrial and ventricular leads were pulled back under fluoroscopic guidance. A new left coronary sinus lead was placed. Next, right atrial (RA) and right ventricular (RV) leads were placed and access was obtained via the left coronary sinus as the left subclavian vein was found to be occluded with formation of collaterals. The leads were sutured in fascial planes via silk suture. The pacing and sensing parameters were confirmed. BIV-ICD was placed in the pocket, and the pouch was closed via vicryl sutures. The patient had electrocardiogram (EKG) (Figure 1) and chest X-ray (Figure 2) performed after the procedure. He had developed small pneumothorax from the procedure, which resolved on its own.

**Figure 3:**
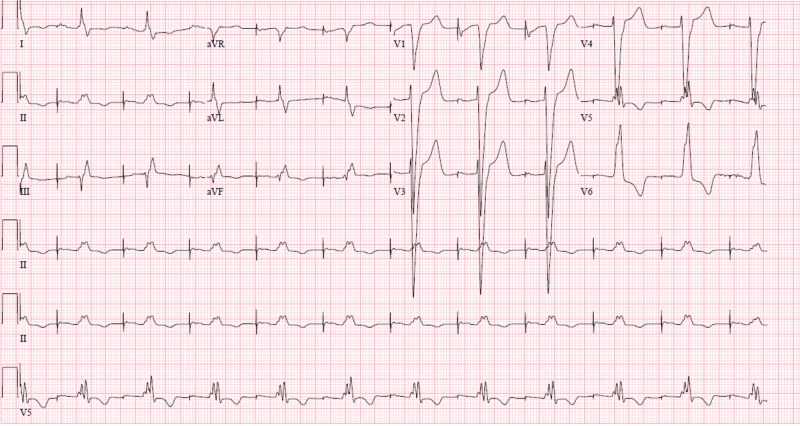
Atrial paced rhythm, loss of ventricular pacing.

**Figure 4:**
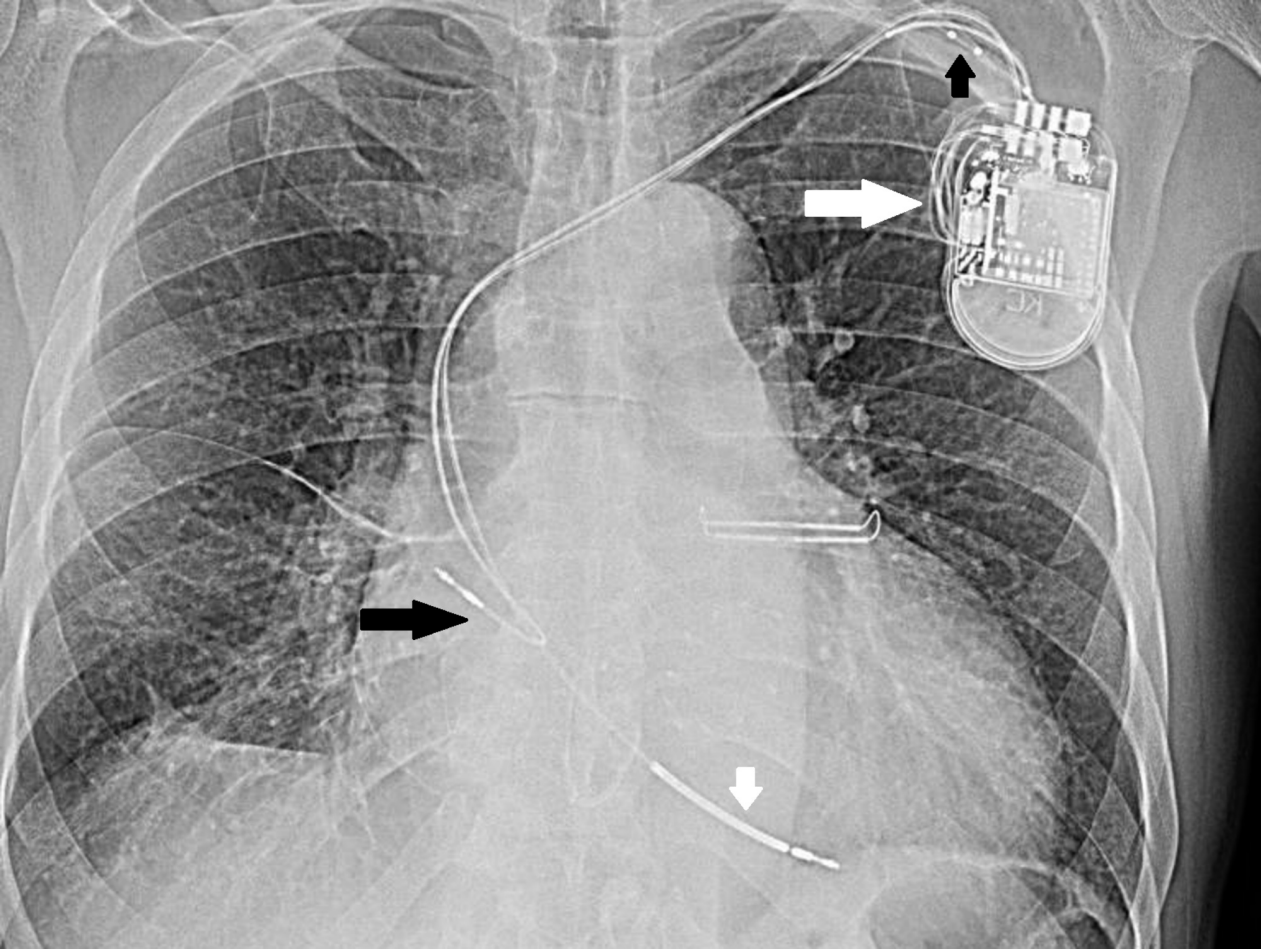
Long white arrow shows spool of wire near device not seen earlier. Small black arrow shows coiled up left coronary sinus lead, small white arrow points to pulled up RV lead, long black arrow shows pulled up RA lead.

**Figure 5:**
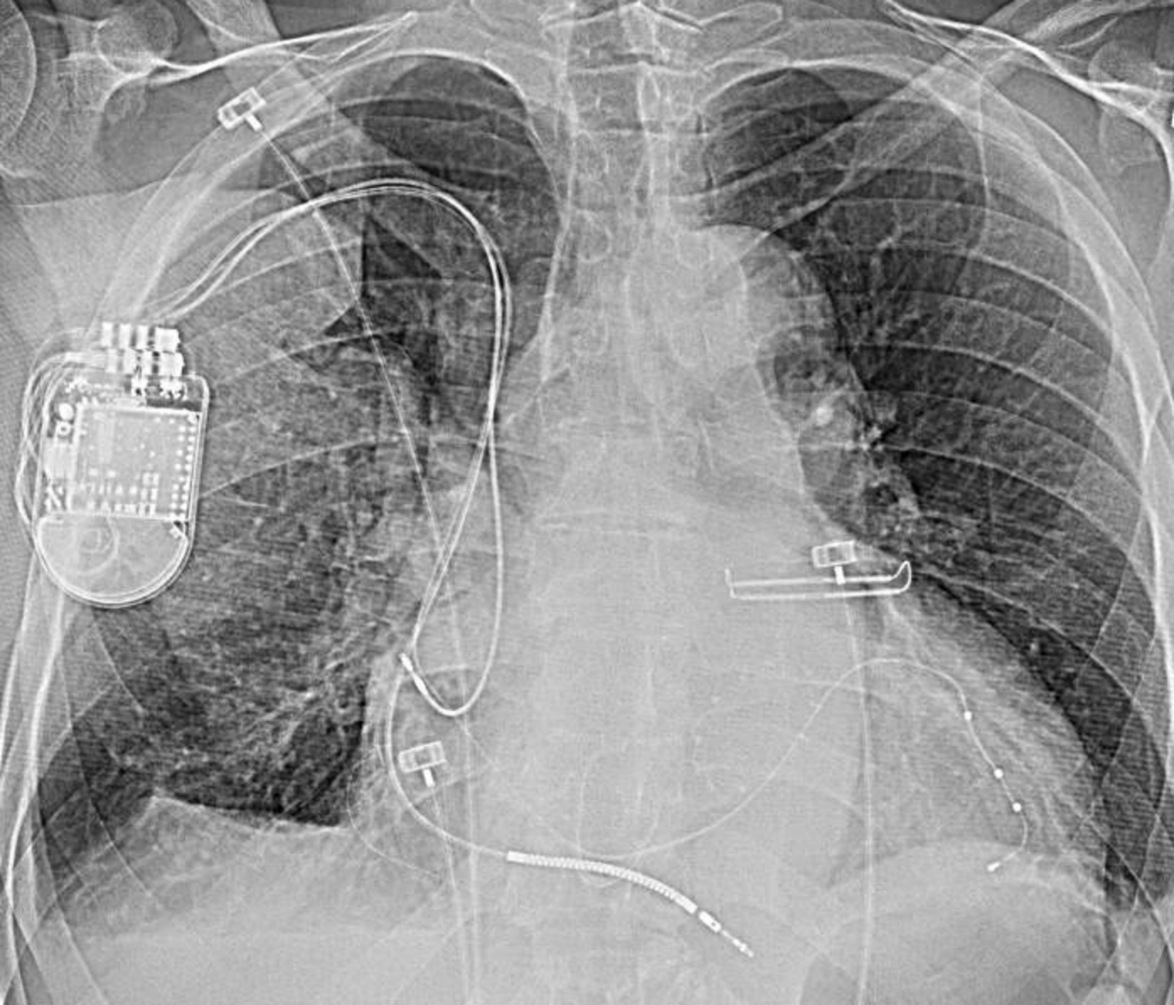
Figure shows device on the right side and leads in the appropriate position.

**Figure 6:**
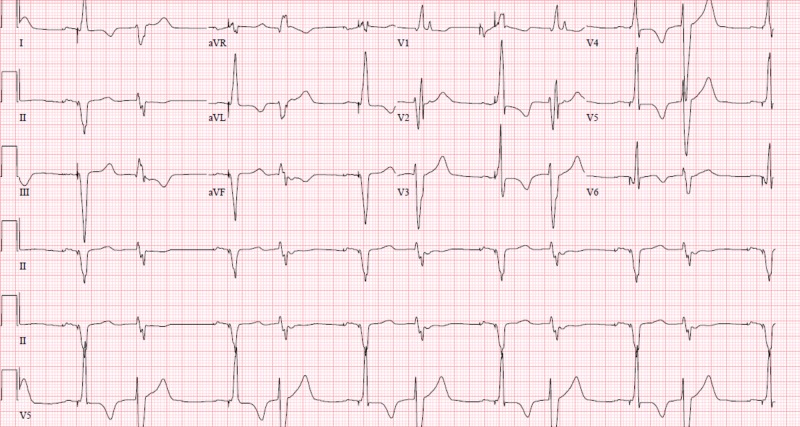
Appropriate biventricular pacing.

The patient was asymptomatic for the next seven months and then he developed increasing shortness of breath. At that point, the EKG (Figure 3) revealed loss of appropriate pacing function. It was discovered that the patient kept massaging his pacemaker pocket site in the last seven months but he denied manipulating the device itself. He had also been moving his arm up and down frequently, which would have led all his device leads to be pulled up towards the device in a ratchet-like fashion. The patient was scheduled again for revision of BIV-ICD and on incision of the generator pocket, the left ventricular lead was found coiled up in the pocket beside the generator (Figure 4). A new left ventricular lead was placed in the coronary sinus and then access was obtained via the right subclavian vein for right-sided leads. His right atrial and right ventricular leads were also found pulled up. A decision was made to place new right atrial and right ventricular leads. It is to be noted that all his device leads were undamaged and his device was found to be in the pocket in normal orientation. After securing the leads via sutures, the pacing and sensing function was analyzed. The pocket was revised and closed with vicryl sutures and the device was sewn to the pectoral fascia. A chest X-ray (Figure 5) and EKG (Figure 6) were obtained after the procedure to ensure the absence of pneumothorax and to document the appropriate functioning of the device, respectively.

## Discussion

Twiddler's syndrome is characterized by pacemaker lead displacement due to rotation of the generator along its long axis, usually occurring in devices loosely placed in the pocket leading to lead breakdown or insulation leakage, thus making it necessary to replace the leads. On the other hand, reel syndrome is characterized by the rotation of the generator along the transverse axis leading to the coiling of the leads by conscious or unconscious manipulation of the device, however, without any damage to the leads. Ratchet syndrome involves upward and downward shoulder motion with weak suture sleeve fixation and surplus of leads in the generator pocket [2-6]. The well-known risk factors for twiddler's syndrome are female gender, large device pocket, elderly patients, and history of dementia [7-9].

These phenomena usually present as syncope, arrhythmias, acute or sub-acute exacerbation of heart failure, and/or twitching in the shoulder/chest [9-11]. Usually reel syndrome and ratchet syndrome present early after implantation of the device as in a few weeks to one month, but twiddler's syndrome presents later over a period of months to a year [4]. It is common practice to get a chest X-ray after the device is placed and at the time of presentation to see lead dislodgement. Electrocardiogram and device interrogation are also performed routinely for diagnostic purposes [9].

Due to the dangerous complication of lead displacement or dislodgement, important prevention strategies to address them have been described in the literature. The leads can retract spontaneously without any manipulation to the device by the patient, as in ratchet syndrome, so the leads should be firmly secured in the device pocket via a suture sleeve to prevent retraction [12]. It is important to apply appropriate amount of tension so as not to damage the lead itself but making sure that it is firmly secured [10]. Smaller device pockets, suturing of the device to the fascia, and device placement in the pectoral tissue are also considered important preventative strategies [10]. Last but not the least is patient education to not manipulate the device in any manner [9].

## Conclusions

Our case highlights the importance of early detection of twiddler's syndrome utilizing appropriate diagnostic modalities and suggests that ratchet syndrome can coexist with twiddler's syndrome.
